# Analysis of Drug Effects on iPSC Cardiomyocytes with Machine Learning

**DOI:** 10.1007/s10439-020-02521-0

**Published:** 2020-05-04

**Authors:** Martti Juhola, Kirsi Penttinen, Henry Joutsijoki, Katriina Aalto-Setälä

**Affiliations:** 1 Faculty of Information Technology and Communication Sciences, Tampere University, Tampere, Finland; 2 Faculty of Medicine and Health Technology, Tampere University, Tampere, Finland; 3 Heart Center, Tampere University Hospital, Tampere, Finland

**Keywords:** Drug effect, Induced pluripotent cardiomyocyte, Calcium transient signal, Machine learning, Classification

## Abstract

Patient-specific induced pluripotent stem cell-derived cardiomyocytes (iPSC-CMs) offer an attractive experimental platform to investigate cardiac diseases and therapeutic outcome. In this study, iPSC-CMs were utilized to study their calcium transient signals and drug effects by means of machine learning, a central part of artificial intelligence. Drug effects were assessed in six iPSC-lines carrying different mutations causing catecholaminergic polymorphic ventricular tachycardia (CPVT), a highly malignant inherited arrhythmogenic disorder. The antiarrhythmic effect of dantrolene, an inhibitor of sarcoplasmic calcium release, was studied in iPSC-CMs after adrenaline, an adrenergic agonist, stimulation by machine learning analysis of calcium transient signals. First, beats of transient signals were identified with our peak recognition algorithm previously developed. Then 12 peak variables were computed for every identified peak of a signal and by means of this data signals were classified into different classes corresponding to those affected by adrenaline or, thereafter, affected by a drug, dantrolene. The best classification accuracy was approximately 79% indicating that machine learning methods can be utilized in analysis of iPSC-CM drug effects. In the future, data analysis of iPSC-CM drug effects together with machine learning methods can create a very valuable and efficient platform to individualize medication in addition to drug screening and cardiotoxicity studies.

## Introduction

Induced pluripotent stem cell-derived cardiomyocytes (iPSC-CM) have been utilized to investigate several cardiac diseases.^16^ Function of calcium cycling is essential in the excitation–contraction coupling of cardiomyocytes and calcium cycling studies of iPSC-CMs can give new insight of disease pathology, prevention and treatment. Different studies have shown that calcium transient analysis of iPSC-CMs can be utilized for assessing compounds on cardiac contractility and evaluating drug responses.^17^,^18^

Machine learning together with calcium transient signals of iPSC-CMs can also be utilized in the context of drug research for cardiac diseases. It has been used for the analysis of mechanistic action of drugs in cardiology^12^ and also for electrophysiological influence of chronotropic drugs.^5^ However, thus far, the use of machine learning to analyze and model large sets of calcium transient signals originating from iPSC-CMs seems to be still rare. Previously, we have studied the use of machine learning for the differentiation of normally and abnormally cycling calcium transient profiles or signals of iPSC-CMs on the basis of the recognized and classified peaks in those transient signals.^7^ Cardiomyocytes with normal calcium transients were more frequent (85-90%) for cardiomyocytes of control, healthy individuals (wildtype, WT), but both normal and abnormal calcium transients were roughly equally frequent for diseased cardiomyocytes.^7^^–^9 In addition, we found that it is possible to separate different genetic cardiac diseases from each other and from healthy controls by applying these transient signals: transient signals of one cardiac disease differed from those of another disease or controls.^8^,^9^

In the current research, we applied machine learning for peaks identified from our calcium transient signal data of our previously published drug study of catecholaminergic polymorphic ventricular tachycardia (CPVT)-specific iPSC-CMs.^14^ CPVT is an inherited arrhythmogenic disorder, which is caused by genetic mutations affecting proteins, e.g. cardiac ryanodine receptor (RyR2), that regulate the calcium cycling in cardiomyocytes.^11^,^15^ Here the peaks identified from CPVT specific cardiomyocyte calcium transient signals formed our data input to various machine learning methods in order to study the effect of adrenaline, an adrenergic agonist and dantrolene, an inhibitor of sarcoplasmic calcium release having antiarrhythmic effects.^14^ We ran machine learning methods for iPSC-CM calcium transient data gained from three types of measurement conditions: (1) baseline condition reflecting the spontaneous beating of cardiomyocytes, (2) adrenaline condition, where cardiomyocytes were exposed to adrenergic agonist to increase their beating rhythm and (3) dantrolene condition, where cardiomyocytes, which showed calcium transient abnormalities during adrenaline perfusion were exposed to dantrolene together with adrenaline.

## Material

### Generation of hIPSC-CMs and Their Characterization

This study was approved by the Ethics Committee of Pirkanmaa Hospital District regarding culturing and differentiating of human iPSC lines (R08070). All experimental methods related to hiPSC-CMs have been described earlier.^14^ Briefly, studied iPSC cell lines included six CPVT lines generated from CPVT patients carrying RyR2 mutations including exon 3 deletion, and point mutations P2328S, T2538R, L4115F, Q4201R and V4653F. The iPSCs were differentiated into spontaneously beating CMs using the END2 differentiation method^13^ and dissociated to single-cell level for calcium imaging studies, which were conducted with spontaneously beating Fura-2 AM (Invitrogen, Molecular Probes) loaded CMs. Calcium transient signals were measured with inverted IX70 microscope with a UApo/340 x20 air objective (Olympus Corporation, Hamburg, Germany) with an ANDOR iXon 885 CCD camera (Andor Technology, Belfast, Northern Ireland) and a Polychrome V light source by a real time DSP control unit and TILLvisION or Live Acquisition (TILL Photonics, Munich, Germany) softwares. Calcium signals were acquired as the ratio of the emissions at 340/380 nm wavelengths, and background noise was subtracted before further processing. For drug studies, the changes in calcium were recorded during spontaneous baseline beating, spontaneous beating after exposure to 1 *µ*M adrenaline and spontaneous beating after exposure to 1 *µ*M adrenaline together with 10 *µ*M dantrolene (Sigma). If calcium transient abnormalities were detected after exposure to adrenaline, cells were exposed to dantrolene to see the potential antiarrhythmic response.

### Data Computed from Calcium Transient Signals

We have designed and implemented a computational method to recognize beats or peaks of calcium transient signals originating from iPSC-derived cardiomyocytes.^7^^–^9 The recognition of peaks was based on the computation of first derivative using successive short segments of a few samples from the beginning of a transient signal to its end. When first derivative values increased from roughly zero values rapidly to positive values the beginning of a peak was met, then decreasing first derivative values back close to zero its maximum was found and finally after rapid change to negative values again close to zero the end of the peak was observed. Very small peaks containing smaller amplitudes approximately less than 8% compared with those of the large amplitude peaks in the signal were left out as potential noise.

A biotechnology expert determined whether an iPSC-derived cardiomyocyte had generated normally or abnormally beating cycles. This was also mainly applied in the present study, since this is our first case to apply machine learning methods to drug research with calcium transient signals and we wanted to be as sure as possible with regard to decisions to which type each transient signal should be labelled being central for creating highly qualified training sets for machine learning tests.

**Figure 1:**
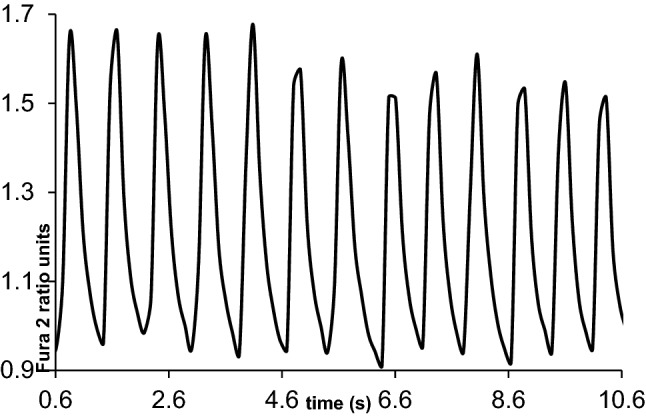
A baseline calcium transient signal segment of around 10 s measured from an iPSC-derived cardiomyocyte in association with CPVT disease. The peaks were detected by the signal recognition algorithm evaluating all of them to be rather normally shaped calcium peaks close the similar size.

**Figure 2:**
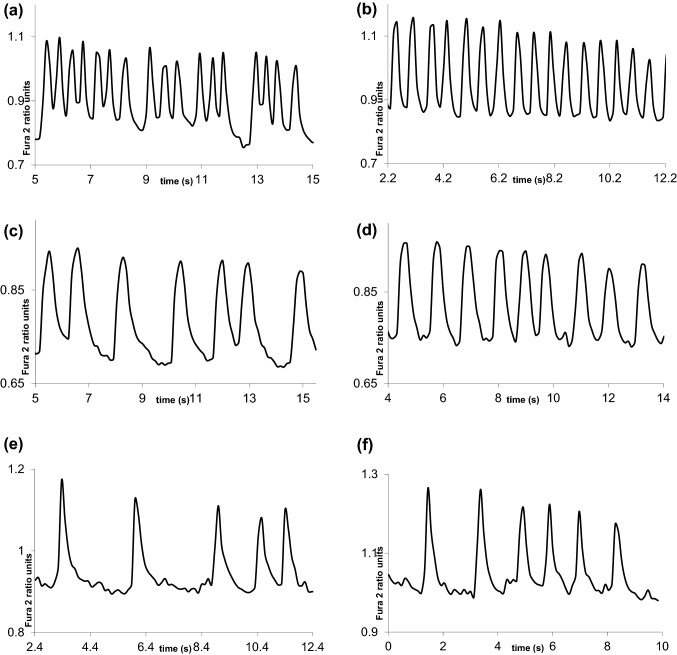
(a) An adrenaline exposure signal segment and (b) its dantrolene responder signal including regular peaks of a roughly similar size, (c) an adrenaline exposure signal segment and (d) its semi-responder containing slight irregularity, and (e) an adrenaline exposure signal segment and (f) its non-responder containing rather similar irregularity as before dantrolene.

We used the data computed from six CPVT cell lines altogether containing 128 calcium transient signals for each of the baseline, adrenaline and dantrolene conditions. The biotechnology expert labelled the signals affected by dantrolene to three classes called responder, semi-responder and non-responder. In a responder signal dantrolene abolished all the calcium cycling abnormalities, which therefore included only normal calcium peaks. In a semi-responder signal dantrolene reduced abnormalities by more than 50% causing the signal to comprise of some abnormally shaped calcium peaks. In a non-responder signal dantrolene reduced abnormalities by less than 50% causing the signal to consist of clearly abnormally shaped calcium peaks. Figures 1 and 2 show example transient signal segments. Table 1 presents their numbers for six CPVT cell lines. In Table 1, cardiomyocytes were first treated with adrenaline and then the effect of dantrolene was analyzed. Only those cardiomyocytes with adrenaline-induced arrhythmias were studied. Cell lines 1, 2 and 4 were mainly responders and most arrhythmias were abolished with dantrolene. Cell lines 5 and 6 were mostly non-responders and dantrolene did not abolish the arrhythmias. Cell line 3 had equal amount of responders, semi-responders and non-responders.

**Table 1:** Numbers of responder, semi-responder and non-responder signals after affecting by dantrolene when 15, 30, 17, 31, 22 and 13 transient signals were measured from six CPVT cell lines, and numbers of recognized peaks.

Cell line (mutation)	Responder transient signals		Semi-responder transient signals		Non-responder transient signals	
	Number of signals	Number of peaks	Number of signals	Number of peaks	Number of signals	Number of peaks
1 (exon 3 del)	12	61	0	0	3	22
2 (P2328S)	21	271	8	116	1	14
3 (T2538R)	6	59	4	42	7	69
4 (L4115F)	16	183	10	138	5	77
5 (Q4201R)	3	32	4	31	15	175
6 (V4653F)	1	9	2	16	10	120
Sum	59	615	28	343	41	477

### Data Computed from Peaks of Calcium Transient Signals

**Figure 3:**
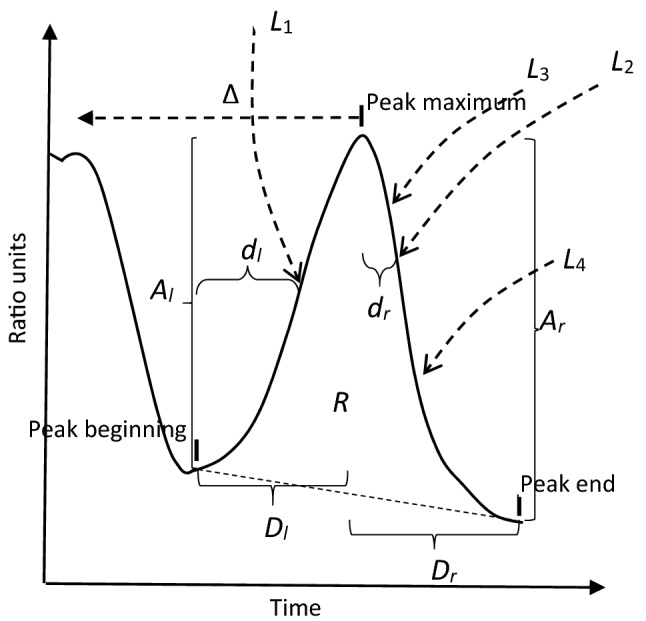
Peak amplitudes *A*_*l*_ and *A*_*r*_, durations *D*_*l*_ and *D*_*r*_, approximate location *L*_1_ for the computation of max(*s*’) (first derivative), approximate location *L*_2_ for |min(*s*′)|, approximate location *L*_3_ for |min(*s*′′)| (second derivative) and approximate location *L*_4_ for max(*s*′′), surface area *R*, duration Δ from the preceding peak, duration *d*_*l*_ from the peak beginning to *L*_1_, and duration *d*_*r*_ from the peak maximum to *L*_2_.

In our data there were baseline signals, adrenaline signals and dantrolene (responder, semi-responder or non-responder) signals, 128 of them in each of three sets. In order to enable an analysis subject to their relations, we first computed variable values from all their valid peaks recognized in the preceding phase. Their computation had been presented in detail previously.^7^^–^9 They are illustrated in Fig. 3. We applied 12 different peak variables^9^: amplitudes *A*_*l*_ and *A*_*r*_ of peak left and right sides, their durations *D*_*l*_ and *D*_*r*_, their maximum max(*s*’) (from peak left side) and absolute minimum |min(*s*’)| (from peak right side) for the first derivative *s*’, maximum max(*s*’’) and absolute minimum |min(*s*’’)| of the second derivative *s*’’ from the peak right side, peak surface *R* area between the peak curve and the line between the peak beginning and end, duration Δ from the peak maximum back to that of the preceding peak or, if this non-existent, back to the beginning of the signal, duration *d*_*l*_ from the peak beginning to the location of the first derivative maximum (inside the left peak side), and duration *d*_*r*_ from the location of the peak maximum to the location of the first derivative absolute minimum (inside the peak right side).

In Table 2 the means of the results differ in most cases if we compare variable by variable between all possible pairs of baseline, adrenaline and dantrolene response, semi-response and non-response. Nevertheless, there are also some such pairs in which differences are small. Frequently standard deviations are relatively great compared to the means in the same cells. All 12 variables are used jointly in actual machine learning analysis Thus, the differences of the means and standard deviations of the single variables do not predict inevitably how effectively four classes of adrenaline vs. dantrolene response, semi-response and non-response could be separated from each other. The machine learning analysis is considered in the following section.

**Table 2:** Means and standard deviations of peak variables for baseline, adrenaline and dantrolene signals (responder, semi-responder and non-responder): amplitudes *A*_*l*_ and *A*_*r*_, durations *D*_*l*_ and *D*_*r*_, maximum and absolute minimum of *s*′, maximum and absolute minimum of *s*′′, peak area *R*, time difference, and durations *d*_*l*_ and *d*_*r*_. Note that before the peak recognition the amplitude values in all signals were multiplied by 1000 compared with those in Figs. 1 and 2.

Variables	Baseline	Adrenaline	Responder	Semi-responder	Non-responder
*A*_*l*_	277 ± 181	252 ± 171	236 ± 168	257 ± 174	163 ± 133
*A*_*r*_	279 ± 184	253 ± 171	239 ± 170	260 ± 172	165 ± 134
*D*_*l*_ [s]	0.212 ± 0.109	0.213 ± 0.107	0.247 ± 0.097	0.243 ± 0.099	0.228 ± 0.106
*D*_*r*_ [s]	0.438 ± 0.303	0.397 ± 0.247	0.424 ± 0.229	0.413 ± 0.136	0.347 ± 0.219
max(*s*’)	1731 ± 1124	1653 ± 1243	1451 ± 1037	1374 ± 1035	1231 ± 1284
|min(*s′*)|	1005 ± 592	967 ± 633	848 ± 485	820 ± 465	730 ± 703
max(*s*′′)	3852 ± 3098	3983 ± 3887	3405 ± 2657	2606 ± 2030	3665 ± 4807
|min(*s*′′)|	2883 ± 3450	2973 ± 3752	2483 ± 2802	1758 ± 2081	2463 ± 3133
*R*	89.1 ± 76.2	76.7 ± 64.9	82.7 ± 102.4	100.0 ± 76.2	49.8 ± 45.9
Δ [s]	1.038 ± 0.603	0.672 ± 0.440	1.082 ± 0.443	1.017 ± 0.267	0.674 ± 0.421
*d*_*l*_ [s]	0.128 ± 0.086	0.132 ± 0.084	0.160 ± 0.085	0.143 ± 0.083	0.145 ± 0.088
*d*_*r*_ [s]	0.098 ± 0.075	0.096 ± 0.071	0.103 ± 0.066	0.125 ± 0.087	0.092 ± 0.069

### Classification Methods Applied to Separation of Signal Classes

Generally speaking, in machine learning there are three issues that often are the most critical ones related to the success of applying machine learning methods to real world problems. These issues are selecting the right variables, constructing training and test sets properly for the machine learning methods and finding the right hyperparameter values for the algorithms. When these issues are solved with respect to the problem handled, in many cases the results are good or satisfactory at least. For the separation of the transient signal classes we used all the 12 variables consistently with all classification algorithms.

The classification or separation among five different calcium signal classes (baseline, adrenaline, responder, semi-responder and non-responder) is based on several machine learning algorithms. Testing of several classification algorithms is necessary in practice in order to obtain as wide empirical evidence as possible which algorithm would be the most suitable for the application considered. However, we need to remember that the application examined in this paper includes several research lines, which are covered in the following section in a more detailed way. Since the machine learning research in this paper is application oriented by nature, each one of the research lines requires a separate and detailed analysis and the analysis consists of in this case the selection of the most suitable classification algorithm and hyperparameter values, if necessary. Because all research lines have their own special characteristics such as different dataset and/or class distribution between each other, we cannot guarantee that there is only one classification algorithm, which would outperform other methods tested in all possible research lines. Machine learning is in practice tailoring algorithms to work in a specific domain. The best results presented in the next section are directional for researchers and/or practitioners who work with the same kind of research problem as described in this paper. The results give perspective which methods would be the best ones for the similar research problems what one is examining. Fine-tuning of an algorithm like selecting the optimal hyperparameter values are always data and problem dependent so there is not any clear guidelines how to select the values optimally.

The methods tested are the same as in our earlier studies.^8^,^9^*K*-nearest neighbor nearest searching (KNN) was applied with Chebychev metric, with cityblock (Manhattan) metric, with correlation measure, with cosine measure, with Euclidean metric, with Mahalanobis measure, with standardized Euclidean metric and with Spearman measure. All of them were tested with equal, inverse or squared inverse weighting, naturally, and all the measure and weighting combinations were tested with odd *K* values from 1 to 37 (number of nearest neighbors searched). The selection of odd *K* values is justified on the basis of tie exclusion that may happen if even *K* value is used.

**Figure 4:**
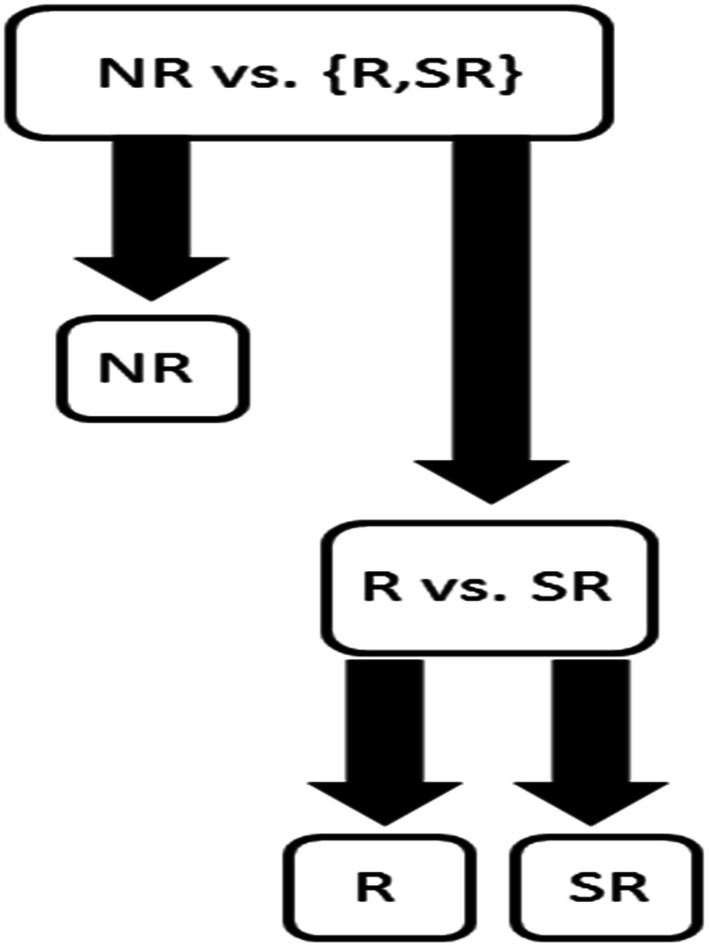
Hierarchical LS-SVM structure used to separate non-responder (NR), responder (R) and semi-responder (SR). Each inner node in the structure consists of a binary LS-SVM classifier.

Besides KNN algorithm, linear, quadratic and Mahalanobis discriminant analysis were examined as well as decision trees (CART), multinomial logistic regression (logistic regression in binary case), Naïve Bayes with normal distribution, Naïve Bayes with kernel density estimation and with the normal, box, Epanechnikov and triangle kernels were examined. Random forests were also investigated and the number of trees tested in a forest ranged from 1 to 100 with step size of 1. When a random forest has only one tree in a forest, the tree structure differs from the tree structure given by the CART algorithm. Furthermore, least squares support vector machines (LS-SVMs) were used with the linear, quadratic, cubic, and radial basis function (RBF) kernels. Test set-ups included both binary and multi-class classification schemes and, hence, with LS-SVMs also a multi-class extension of LS-SVM was required. In this study a hierarchical approach of LS-SVM was used that was similar to a method applied in Ref. 6 More specifically, Fig. 4 represents the hierarchical LS-SVM used in this study. With LS-SVMs boxconstraint (*C*) and *σ* parameter (encountered in RBF kernel) had the same parameter value space of {2^−12^, 2^−11^,…, 2^17^}.

In other words, the polynomial kernels were tested with 30 different values of C and RBF kernel with 900 (*C*,*σ*) combinations.

Now the variables and tested hyperparameter values are explained from the three essential issues with respect to machine learning algorithms. In this paper we used leave-one-out (LOO) method in a signal level for classification. Here, in each LOO round the data from one signal is left for test set and the rest of the data forms a training set. We must also notice that the data of one signal is a collection of data derived from the peaks within the signal. Hence, every row in a test set corresponds to a data gained from one peak within a signal. Since we are dealing with signal classification, we need to separate signal level and peak level information from each other. A classification algorithm forms a model based on peak level information and gives prediction for each row (peak) in a test set. However, the transformation from peak level prediction into signal level prediction needs to be made in order to achieve the signal level classification result. This transformation is made based on majority voting method. In other words, we take the mode of test set predictions (test set consists of only data from one signal) to gain the signal level classification. Since mode can be unambiguously determined (for example, test set includes 10 rows of data and 5 rows obtains class A as predicted class label and the rest 5 rows obtains class B as a predicted class label), a strategy for ties must be developed. In this paper we used the same strategy as in Refs. 7 and 9 to solve the ties, so a reader can find the detailed description about the tie solving strategy from the given references. After LOO procedure, we have obtained a signal level prediction for each signal in a dataset. Hence, we can compare the predicted class labels to ground truth class labels and define a confusion matrix. From the confusion matrix we evaluate accuracy (trace of confusion matrix divided by the sum of elements in a confusion matrix) and other evaluation measures (true positive, TP). When a classification algorithm required parameter tuning, we performed LOO procedure with all parameter values tested and selected parameter value that gained the highest accuracy. The reason behind the use of LOO is the lack of data. Machine learning algorithms usually require a lot of data to work well and to produce a reliable model for prediction. With the use of LOO we maximized the size of training data. Before any classification, we applied *z*-score standardization to the whole dataset in order to obtain all variables equally important.

### Classification Results

In the following, only the best classification method is mentioned for every test set-up. The performance was evaluated by applying true positive, false positive and negative in confusion matrices, and classification accuracy as usual. First, we classified among three dantrolene transient signal classes to study how well these can be separated from each other. Table 3 where correctly classified are presented along the diagonal presents the best results generated by random forests with 14 trees and giving classification accuracy of 65.6% and sensitivities (true positive rates) of 79.7% for responders, 35.7% for semi-responders and 65.9% for non-responders.

**Table 3:** Classification of three dantrolene classes: responders (R), semi-responders (SR) and non-responders (NR).

True class	Predicted class		
	R	SR	NR
R	**47**	3	9
SR	12	**10**	6
NR	12	2	**27**

True positive cases are written in Bold

According to Table 3 semi-responders were classified worse than the other two. The results in Table 3 denote that the two classes of responders and semi-responders may resemble somewhat each other, because more semi-responders (12 signals) were incorrectly classified into the class of responders than correctly to semi-responders (10 signals). Furthermore, on the basis of Table 1 the number 28 of semi-responder signals being the minority class here is less than 59 and 41 of responders and non-responders. The poor results of semi-responders may partly be caused by their characteristics of being between the responders and non-responders as Fig. 2 showed.

We united the responders and semi-responders (RSR) and then computed results given in Table 4. Accuracy is now 78.9%, when sensitivities are 90.8% for RSR and 53.7% for NR produced by random forests with 36 trees. Here the quite poor sensitivity of the non-responders might be inflicted by their minority in the data, 32% of all.

**Table 4:** Classification of dantrolene classes: responders merged with semi-responders (RSR), and non-responders (NR).

True class	Predicted class	
	RSR	NR
RSR	**79**	8
NR	19	**22**

True positive cases are written in Bold

Next we studied classification when the semi-responder and non-responder signals are merged. In Table 5 their results are shown producing the accuracy of 73.4% with sensitivities of 69.5% for responders (*R*) and 76.8% merged semi-responders and non-responders (SNR) by *K*-nearest neighbor searching algorithm with city block metric and equal, inverse or squared inverse weighting, *K* equal to 1 for all these three.

**Table 5:** Classification of dantrolene classes: responders (R), and semi-responders merged with non-responders (SNR).

True class	Predicted class	
	R	SNR
R	**41**	18
SNR	16	**53**

True positive cases are written in Bold

Because the accuracy of the test se-up for Table 4 is higher than that for Table 5, in other words, there are less incorrect predictions (19 + 8) in Table 4 than those (16 + 18) in Table 5, we continued to apply the fusion of responders and semi-responders. Note that the number of these signals was rather limited, thus, not the very best starting point for machine learning tasks.

We continued by classifying adrenaline vs. merged responders and semi-responders (RSR). Their results in Table 6 produced the accuracy of 71.2% and sensitivities of 70.3% for adrenaline (A) and 72.4% for RSR computed with least-squares support vector machines with the radial basis kernel with parameters *C *= 2^10^ and *σ *= 2^2^.

**Table 6:** Classification of adrenaline vs. dantrolene signal classes or responders merged with semi-responders (RSR).

True class	Predicted class	
	A	RSR
A	**90**	38
RSR	24	**63**

True positive cases are written in Bold

Next we computed adrenaline against non-responders shown in Table 7. This achieved the accuracy of 78.1% and sensitivities of 90.6% for adrenaline (A) and only 39.0% for non-responders (NR) given by Naïve Bayes with kernel density estimation and with the triangle kernel. The non-responders might suffer from the minority of 24% only when the majority of the adrenaline class was very predominant in classification. Nevertheless, the main reason of the low sensitivity of NR is that the non-responders resemble more or less the adrenaline signals. This is quite natural, because then dantrolene had not influence, in other words, it did not correct peak shapes in these NR signals.

**Table 7:** Classification of adrenaline vs. dantrolene class non-responders (NR).

True class	Predicted class	
	A	NR
A	**116**	12
NR	25	**16**

True positive are written in Bold

We also computed others such as merging semi-responders and non-responders and tested with that and also with three separate dantrolene classes against adrenaline, but these gave somewhat poorer results than the presented above.

We still compared the situation between baseline and adrenaline transient signals. Results are shown in Table 8 and were computed with least squares support vector machines with the radial basis kernel with parameters *C *= 2^−10^ and *σ *= 2^2^. Accuracy is 54.7% and sensitivities are 57.0% for adrenaline signals (A) and 52.3% for baseline signals (B). These are rather low close to 50% indicating that A and B do not differ much from each other. This is in line with the result in our previous publication where we showed that CPVT-CMs demonstrated marked amount of calcium transient abnormalities both in baseline and in response to adrenaline.^14^

**Table 8:** Classification of baseline (B) vs. adrenaline (A)

True class	Predicted class	
	B	A
B	**73**	55
A	61	**67**

True positive cases are written in Bold

## Discussion

In drug development industry, cardiotoxicity is one of the leading causes of failure for a new therapeutic molecule.^1^ Another issue is the efficacy of new potential molecules.^4^ Currently, pharmaceutical industry relies upon animal testing and genetically transformed non-cardiac cells lines^2^,^3^ but iPSC-CMs could offer more physiological drug testing model mimicking human myocardium. Machine learning together with calcium transient signals of iPSC-CMs can provide more accurate and faster pre-clinical detection method as well as in-depth details of cell behavior in the context of drug research for cardiac diseases. In recent years more research has focused on creating new screening platforms for iPSC-CMs for disease modeling and drug responses, even in a high throughput level.^17^,^18^ At some point in the near future this will result in large multidimensional datasets, which requires improved automated and comprehensive analysis methods. For a researcher the analysis of data often requires some simplifying of the gained data for example a limited number of analyzed parameters of complex dataset. With machine learning a dataset can be handled without losing information in the analysis process. Therefore, machine learning is an effective method to be exploited in drug studies, which can even define and predict drug responses.

Here we showed how machine learning can provide insights in the detection of drugs affecting calcium cycling properties of iPSC-CMs. We may assess that the results obtained are good, but not excellent. Merging responder and semi-responder dantrolene signals as made in Table 4 and classifying against non-responders gave the very good classification accuracy of 78.9%, but the imbalanced sensitivities of 90.8% for the merged responders and semi-responders and 53.7% for the non-responders. Obviously, the minority class position of the non-responders was slightly unfavorable for classification. The results in Table 6 indicated that responders merged with semi-responder transient signals can also be separated from those of adrenaline with the relatively good accuracy of 71.2% with the balanced sensitivities of classes adrenaline and responders. Instead, adrenaline against non-responders was not so successful in Table 7. On one hand, this showed that often dantrolene changed cardiomyocytes exposed with adrenaline enough classifying them correctly to belong to the class of responders merged with semi-responders (Table 6). On the other hand, the complexity to separate the non-responders from the adrenaline signals is sensible, since then dantrolene affected or changed the properties of adrenaline signals only very little or not at all (Table 7). Nevertheless, returning to Fig. 2 the phenomenon is reasonable, when in Fig. 2 adrenaline has deformed a part of peaks to be abnormal, but next dantrolene in a responder signal of Fig. 2b has affected so that all peaks are normal without irregularity. If dantrolene did not affect this way, the case would be such as in Fig. 2f. Peaks not affected by dantrolene are then mostly abnormal, i.e. quite random as to their size and form.

From the methodological point of view, the results from different classification cases show that Random Forests classifier and Least-Squares Support Vector Machines (binary classifier or tree-based multi-class extension) have gained top accuracies in majority of the classification cases. Hence, these two classification algorithms seem to be suitable for the research problems considered in this paper. Nevertheless, we need to remember that this is a preliminary paper and there exist several other machine learning algorithms which also could be used for the classification problems. However, they are left to future research. For example, deep learning methods such as LSTM networks have obtained a lot of increasing attention and can be used to classify calcium signal classes. Deep learning methods were not used in this paper since the amount of data is still relatively small and deep learning methods require generally a large training set in order to form a reliable predictive model.

It would have been tempting to attempt to classify signals of each cell lines as performed above for all of them together. This would have been interesting when Table 1 showed that the six cell lines contained very different numbers of three dantrolene classes. Three of them were responding, two were not and one was in the middle of these. Unfortunately, the classification of the cell lines separately was not yet possible, because the numbers of signals per a cell line was still so low meaning they were already at their minima, i.e., 128 adrenaline signals and the same total from three dantrolene signal classes, as to the use of machine learning methods. These cannot learn on the basis of very small data sets. This means that adding more data in the future may be promising.

In cardiac field, machine learning has been exploited when studying the effects of β-drenergic drugs on iPSC-CMs with voltage sensitive dye method to assess, classify, and predict membrane depolarization after drug exposure.^5^ In addition, machine learning of cardiac drug effects on contractile force of electrically paced embryonic stem cell derived cardiomyocytes have been studied to create classification model to predict mechanistic actions of an unknown cardioactive drug.^11^ With calcium signaling data, machine learning and classifications have been exploited to evaluate and detect the functional response of calcium release sites in cardiomyocytes^10^ and in neuroscience to predict and classify epileptic seizures.^19^ However, thus far, the use of machine learning to analyze and model drug effects originating particularly from calcium transient signals of iPSC-CMs is new.

In the future, we will collect more data in order to enable the use of machine learning more efficiently. Perhaps, it would be reasonable to utilize only two classes after the use of dantrolene or other drugs, since it might be difficult to determine semi-responder signals even by a human expert as well as by a machine learning program. In any case, the preliminary outcomes given by machine learning encourage us to continue and extend the current research. Machine learning could make personalized medicine become a reality while helping to find a suitable drug, where machine learning could be used for studying the appropriateness of a drug for the treatment of a genetic cardiac disease. It could also provide a human-based platform to study the efficacy of a new molecule as well as cardiotoxicity.

We have shown here that machine learning of calcium signal data is clearly useful for drug research and will probably increase its capability in this purpose when more and more drug response data is gained. In the long term, standardizations of the machine learning methods and the evaluation of drug responses from calcium transient signals are needed. Also, higher amount of calcium signal data will further improve statistical reliability of the drug response analysis. In the future, data analysis of iPSC-CM drug effects together with machine learning methods can create a very valuable and efficient platform for pharmaceutical industry for predicting cardiotoxicity and efficacy and also to personalize medication.
